# Practicable assessment of cochlear size and shape from clinical CT images

**DOI:** 10.1038/s41598-021-83059-6

**Published:** 2021-02-10

**Authors:** Andrew H. Gee, Yufeng Zhao, Graham M. Treece, Manohar L. Bance

**Affiliations:** 1 Department of Engineering, University of Cambridge, Trumpington Street, Cambridge, CB2 1PZ UK; 2 Department of Clinical Neurosciences, University of Cambridge, Level 3, A Block, Box 165, Cambridge Biomedical Campus, Cambridge, CB2 0QQ UK

**Keywords:** Biomedical engineering, Bone imaging, Three-dimensional imaging, Tomography, Bone

## Abstract

There is considerable interpersonal variation in the size and shape of the human cochlea, with evident consequences for cochlear implantation. The ability to characterize a specific cochlea, from preoperative computed tomography (CT) images, would allow the clinician to personalize the choice of electrode, surgical approach and postoperative programming. In this study, we present a fast, practicable and freely available method for estimating cochlear size and shape from clinical CT. The approach taken is to fit a template surface to the CT data, using either a statistical shape model or a locally affine deformation (LAD). After fitting, we measure cochlear size, duct length and a novel measure of basal turn non-planarity, which we suggest might correlate with the risk of insertion trauma. Gold-standard measurements from a convenience sample of 18 micro-CT scans are compared with the same quantities estimated from low-resolution, noisy, pseudo-clinical data synthesized from the same micro-CT scans. The best results were obtained using the LAD method, with an expected error of 8–17% of the gold-standard sample range for non-planarity, cochlear size and duct length.

## Introduction

The size and shape of an individual human cochlea are of profound interest when planning cochlear implant surgery. Knowing the size of the cochlea would enable the clinician to make an informed choice of electrode array and insertion depth, one aim being to preserve any residual, low-frequency natural hearing, which can be beneficial in difficult listening conditions^1,2^. Over-insertion runs the risk of destroying residual hearing, while under-insertion may result in insufficient coverage of the frequency components required for good “electric hearing” through the implant^3^. Additionally, since the cochlea is tonotopically organized, if the electrodes and their assigned frequencies can align with the natural tonotopic arrangement, speech recognition may be enhanced^4^.

Beyond cochlear size, a more nuanced knowledge of cochlear shape would allow the clinician to be forewarned of potentially traumatic insertion contact with the basillar membrane and the lateral wall. The immunological and fibrosis events that follow such trauma may damage the neural structures that respond to electrical stimulation. The nature, force and site of insertion contact will be determined by the shape of the cochlea, particularly in places where the array has to bend^5–8^. Unfortunately, this degree of patient-specific planning is difficult to achieve given the relatively low resolution of current, preoperative computed tomography (CT) imaging. The human cochlea has outer dimensions of approximately $10\,\text {mm} \times 8\,\text {mm} \times 4\,\,\text {mm}$^9^, which corresponds to only $33 \times 27 \times 13$ voxel widths in typical $0.3\,\text {mm}$ clinical CT imaging.

High resolution morphometry of the inner ear is achievable through the use of cadaveric temporal bone specimens, which are either subject to micro-CT imaging with isotropic voxel dimensions of around $20\,\upmu \text {m}$^10^, or used to produce exquisite corrosion casts of the ductal structures which are then photographed and measured^11^. The emphasis of many of these studies is on measurements that are of relevance to cochlear implantation, these falling naturally into three broad categories: measurements of the cochlea’s overall size (total coiling angle, diameter, length, cross-sectional ductal area)^10,12–14^; measurements of the cochlea’s “vertical” trajectory in the direction of the modiolar axis^7,15,16^; and local measurements at the round window that constrain the initial insertion and bending angles of the electrode, when not performing a cochleostomy^17,18^.

Efforts to measure cochlear morphology from low-resolution, clinical CT images fall into two broad categories. Two-dimensional methods attempt to infer cochlear size and shape from a set of discrete measurements taken in specific planes^19,20^. However, Koch et al.^21^ cast doubt on the accuracy of such measurements and suggest that full, three-dimensional analysis is preferable. Falling into this second category is the work of Noble et al.^22^, who built a statistical shape model (SSM) of the cochlea using six micro-CT scans of cadaveric temporal bones, and then assessed how well the SSM could be fitted to low-resolution, clinical scans of five of the same bones. Evaluation metrics were based on Dice similarity coefficients and surface errors, so it is unclear how well this method can estimate surgically relevant parameters. Kjer et al.^23^ developed a similar approach using a statistical deformation model, reporting measurement accuracy and precision for cochlear length, width and height in addition to surface errors, but with no consideration of vertical trajectories. van der Jagt et al.^24^ describe an automatic, three-dimensional tracing method that was used to estimate inner and outer wall radii, duct diameter and vertical trajectory in low-resolution CT scans of 242 patients. Significant variation was observed in the cohort, but there was no validation against gold-standard measurements. Iyaniwura et al.^10^ present a method to fit a grayscale cochlear atlas to low-resolution, clinical CT data using sequential landmark, affine and B-spline registration. Evaluation was performed using 20 specimens scanned at micro-CT and clinical CT resolutions. Gold-standard, micro-CT “A-values”, which correlate to some degree with insertion depth angle^13^ and cochlear duct length^25^, were compared with A-values derived from the fitted atlas and also A-values estimated by experts on the clinical CT images. There was no consideration of vertical trajectories. Heutink et al.^26^ used 123 ultra-high-resolution clinical CT scans to train, validate and test a novel deep learning approach to cochlea localization, segmentation and analysis. Errors were calculated between automatic measurements of cochlear volume, duct length and basal lumen diameter, and corresponding ground truth measurements obtained manually. Again, there was no consideration of vertical trajectories.

In this work, we describe a fast, simple and freely available method to fit surface models of the otic capsule to CT data. The fitting may be directed by a statistical model, in the spirit of Noble et al.^22^ and Kjer et al.^23^, or constrained only by a smoothness criterion, in the spirit of Iyaniwura et al.^10^. We compare the performance of the two approaches, with specific reference to three-dimensional, surgically relevant measurements like those considered by van der Jagt et al.^24^. Particular emphasis is placed on an improved metric for characterizing the different vertical trajectories first described by Avci et al.^15^. Validation is by way of pseudo-clinical CT data synthesized from the original, gold-standard micro-CT images.

## Materials and methods

### Temporal bone specimens and micro-CT scanning

A convenience sample of 18 human temporal bones was provided by the Human Anatomy Centre at the Department of Physiology, Development and Neuroscience, University of Cambridge, who approved their use in this study. All experiments were performed in compliance with the UK Human Tissue Act 2004 (licence no. 12146). The donors had given informed consent for the use of their bodies for anatomical research. The specimens were scanned using a Nikon Metrology XT H 225 ST micro-CT scanner (Nikon Metrology NV, Leuven, Belgium) at $125\,\text {kV}$, $120\,\upmu \text {A}$, 1080 projections, 2 frames per projection and $1\,\text {s}$ exposure time. Reconstruction was at an isotropic voxel resolution of around $25\,\upmu \text {m}$, apart from specimen #18, which was a larger bone section reconstructed at $61\,\upmu \text {m}$. The bones were a mixture of left and right sides and were all unimplanted apart from specimen #17, which was implanted before scanning (as part of a different study) and whose scans therefore suffered significant beam-hardening artefacts.

### Construction of the template and statistical shape models

**Figure 1:**
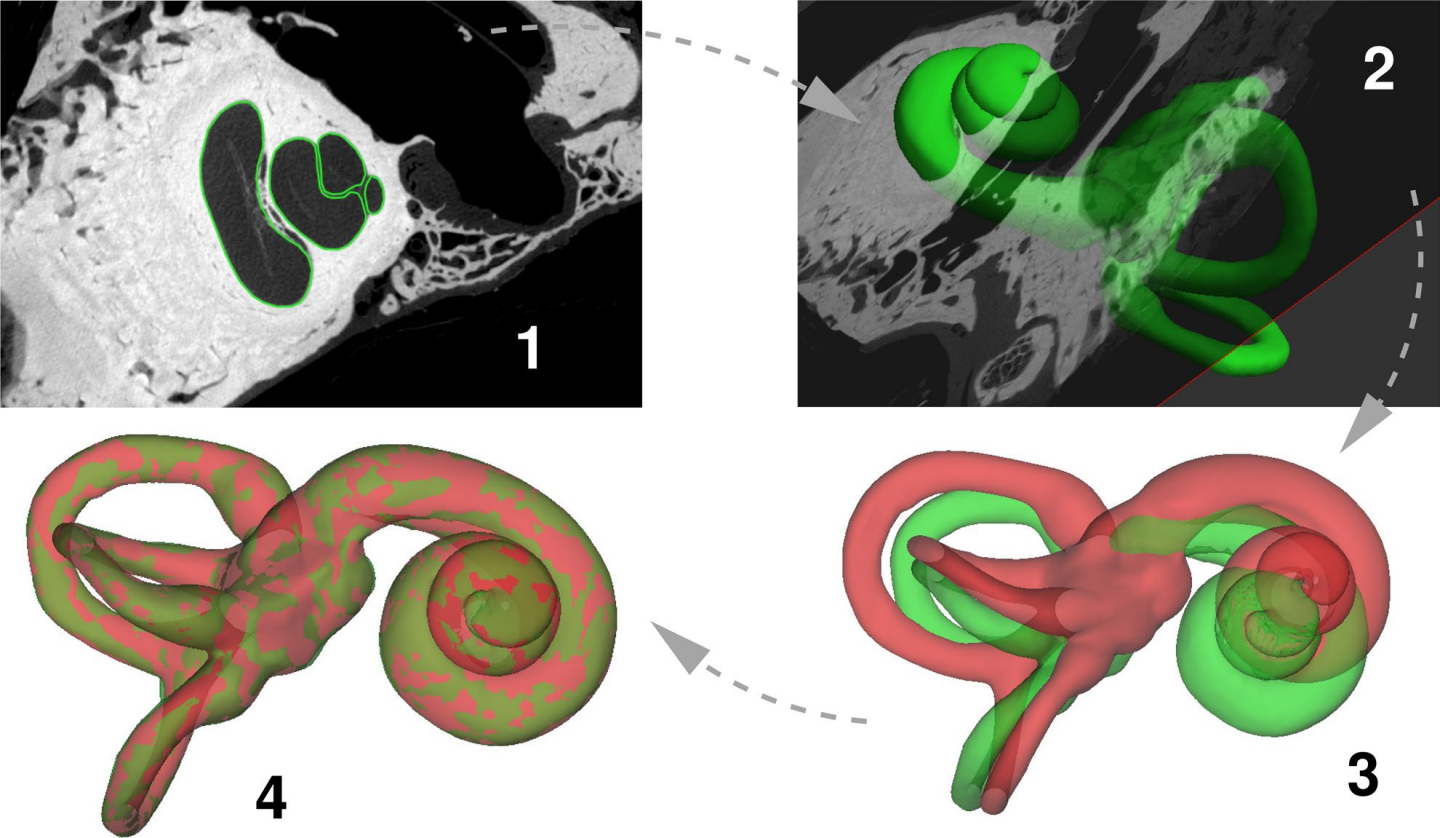
Constructing otic capsule models. (1) The micro-CT scans were segmented in Stradview by simple thresholding followed by manual tidying up of the contours. (2) Stradview was then used to construct triangulated surface meshes of each specimen. (3) Since each mesh has a different number of triangles and vertices, the next step is to align a common template mesh (red) with each specimen (green). This allows statistical analysis of the deformation at each of the template’s vertices, and subsequent construction of an SSM. (4) The alignment involves translation, rotation, isotropic scaling and (if necessary) reflection, followed by a nonrigid thin-plate spline deformation. The deformation was computed in wxRegSurf using the sliding semilandmark algorithm.

Figure 1, steps 1 and 2, show how the 18 micro-CT scans were segmented and the otic capsules represented as triangulated surface meshes. A template surface was then constructed as follows. One of the 18 specimens was selected, by eye, as being the most “average”. The chosen mesh was registered to all 18 specimens, and the mean deformation was calculated and applied at each vertex, producing a mean otic capsule surface. This surface was re-triangulated to a reasonable resolution (11,145 vertices, sufficient to capture the shape without an excessive number of vertices that would only add to the computational complexity of surface registration), the resulting mesh serving as the template for all remaining experiments in this paper. Steps 3 and 4 show how the template mesh was then registered to each specimen using the sliding semilandmark algorithm, originally developed for planar morphometry^27,28^ and subsequently extended to surfaces^29^. Segmentation and mesh construction were performed using Stradview (mi.eng.cam.ac.uk/Main/StradView), while surface registration was carried out in wxRegSurf (mi.eng.cam.ac.uk/~ahg/wxRegSurf).

Following registration, the $n=18$ sets of deformed template vertex coordinates were standardized for location, orientation and scale using Procrustes analysis^30^. This involves translating each specimen to a common origin, scaling to unit centroid size, and then rotating to minimize the sum of the squared distances between the vertices of each specimen and the undeformed template mesh. We then rescaled each specimen’s vertex coordinates by its centroid size, and used principal component analysis to build a point-based SSM from the resulting *n* sets of coordinates. Let $\mathbf{X}_i$ be the 33435-element vector formed by concatenating the coordinates of individual *i*, and let $\hat{\mathbf{X}} = \frac{1}{n} \sum _{i=1}^{n} \mathbf{X}_i$. Then the principal modes of shape variation are the $n-1$ eigenvectors $\mathbf{m}_i$ of the sample covariance matrix $\frac{1}{n-1} \sum _{i=1}^{n} (\mathbf{X}_i - \hat{\mathbf{X}})(\mathbf{X}_i - \hat{\mathbf{X}})^T$ with corresponding non-zero eigenvalues. In SSM-based segmentation, the surfaces of new specimens are encouraged to take anatomically plausible shapes by representing the mesh as a linear combination of the shape modes

1 $$\begin{aligned} \mathbf{X} = \hat{\mathbf{X}} + \sum _{i=1}^{n-1} S_i \mathbf{m}_i \end{aligned}$$

where $S_i$ are referred to as *shape coefficients*. The shape model is available for free download as part of the Stradview package.

### Synthesis of pseudo-clinical CT data

The micro-CT data, and the otic capsules segmented from them, provide the gold-standard measurements for the experiments in this paper. For the clinical measurements, we synthesized pseudo-clinical CT images from the micro-CT data. We achieved this by downsampling the micro-CT until the desired clinical resolution was achieved. We then projected the downsampled data into the CT detector space, in effect recovering the sinogram, added Gaussian noise to the sinogram, and then backprojected the noisy data into the world space. This processing was performed using wxDicom (mi.eng.cam.ac.uk/Main/GMT_wxDicom).

**Figure 2:**
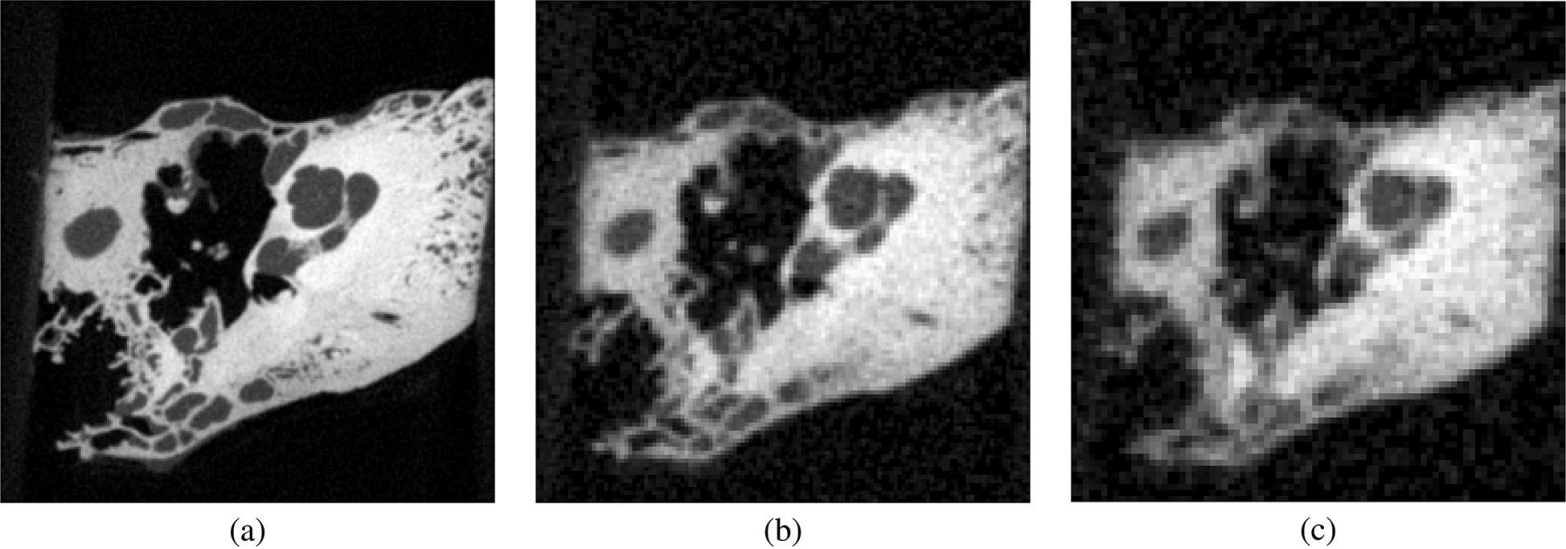
Pseudo-clinical data. (**a**) Next-generation pseudo-clinical CBCT ($0.15\,\text {mm}$ voxels). (**b**) Standard pseudo-clinical MDCT ($0.3\,\text {mm}$ voxels). (**c**) Poor pseudo-clinical MDCT ($0.45\,\text {mm}$ voxels). Note that there is no convenient way to quantify noise levels other than relative to each other. Expressed arbitrarily as the value of wxDicom’s *Detection Noise* slider, the added noise was $60\,\text {dB}$ for the next-generation CBCT data, $70\,\text {dB}$ for the standard MDCT data and $75\,\text {dB}$ for the poor MDCT data.

We synthesized three different classes of pseudo-clinical data, which we shall refer to as standard multidetector CT (MDCT) (isotropic voxel dimension $0.3\,\text {mm}$), poor MDCT (isotropic voxel dimension $0.45\,\text {mm}$) and next-generation cone beam CT (CBCT) (isotropic voxel dimension $0.15\,\text {mm}$): see Fig. 2. The level of Gaussian noise was adjusted by trial and error until the results resembled reference images from the literature. For example, the standard MDCT image in Fig. 2b resembles the exemplar clinical image in Fig. 1c of Phillips et al.^31^, while the poor MDCT image in Fig. 2c is noticeably worse. The next-generation CBCT image in Fig. 2a is superior to those currently found in clinical practice, but resembles the state-of-the-art research images in Zou et al.^32^.

### Fitting the model to CT data

**Figure 3:**
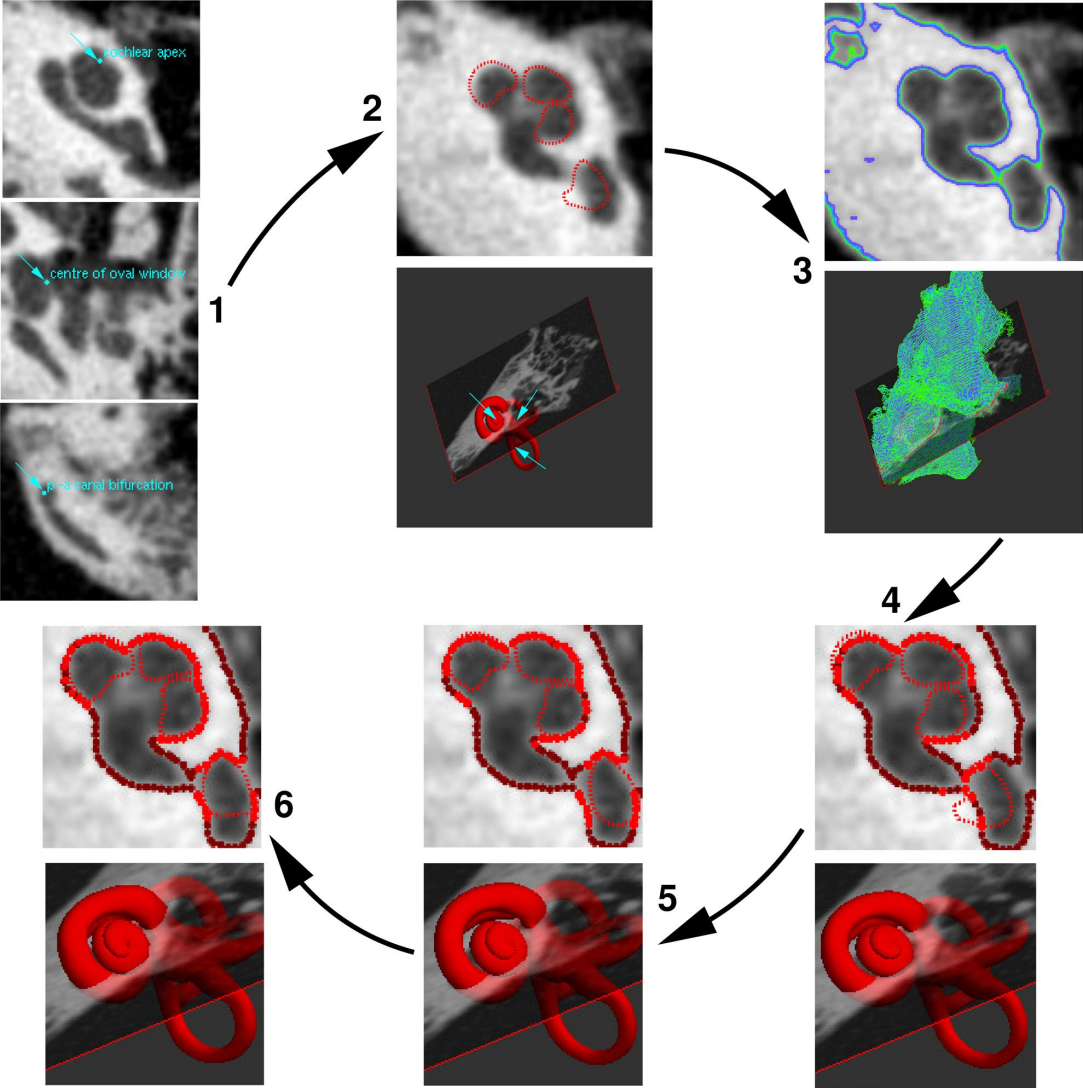
Fitting a model to CT data. (1) The operator scrolls through the axial CT images and places landmarks at the cochlear apex, the centre of the oval window and the posterior-anterior canal bifurcation (at the tip of the crus commune). (2) These landmarks are matched with corresponding, predefined landmarks on the model. An initial, approximate alignment is provided by the similarity transformation (rigid body plus uniform scaling) that minimizes the sum of the squared distances between the three pairs of landmarks. (3) The operator selects a suitable segmentation threshold: this subfigure shows contours thresholded at grayscale 160 (green), 170 (cyan) and 180 (purple). (4) The contour vertices at the selected threshold (170 in this example) provide the point cloud to which the model is fitted. Points closest to a model vertex are displayed in bright red, other points in dark red. (5) Iterative closest point (ICP) registration of the model to the point cloud, with a similarity transformation. (6) Finally, the fit is refined using ICP registration with a nonrigid transformation, either a statistical shape model or a locally affine deformation.

Figure 3 shows the process of fitting the otic capsule model to new CT data. The data in Fig. 3 is pseudo-clinical CT data, though the method is equally applicable to micro-CT data. The model-fitting process is designed to be clinically practicable, in that it requires around 1 min of expert interaction, followed by several minutes of computation.

The first step is to position the template surface at approximately the correct location, by manually identifying three point landmarks in the data: the cochlear apex, the centre of the oval window and the posterior-anterior canal bifurcation (Fig. 3, step 1). Stradview then computes the similarity transformation (rotation, translation and isotropic scaling) that best aligns these three points in the data with corresponding points predefined on the template mesh (Fig. 3, step 2). The operator can optionally reflect the template in the plane of the three points, if the left-right fit was incorrect. The final manual interaction is to select an appropriate grayscale threshold to segment the boundary of the otic capsule (Fig. 3, step 3). The thresholded contours define the point cloud (Fig. 3, step 4) to which the model is now fitted automatically.

An initial, approximate alignment is computed using the iterative closest point (ICP) approach of Besl and McKay^33^. This approximate alignment is parameterized by a second similarity transformation (Fig. 3, step 5). There follows a further iterative process to compute the additional, local displacement of each template vertex (Fig. 3, step 6). Since the thresholded data is noisy (structures other than the otic capsule are captured, and some of the boundaries of the otic capsule, especially at the inner wall and the round and oval windows, are lost), this nonrigid registration must be regularized, to prevent over-fitting of the model to the noise. Stradview offers two methods for regularized, nonrigid registration.

The first is the locally affine registration algorithm of Feldmar and Ayache^34^. Associated with each vertex *k* of the template is a set of neighbouring vertices $N_k$, where each member of $N_k$ lies within a distance *d* of vertex *k*. At iteration *i*, every vertex on the template is paired with the closest point in the cloud. Then, for each vertex *k* on the template, the rigid transformation $\varvec{R}_{k,i}$ is found that minimizes the sum of the squared distances between the transformed vertices in $N_k$ and their partners in the cloud. The local displacement of vertex *k* is then calculated using a proximity-weighted average of all the rigid transformations $\varvec{R}_{k,i}$ within $N_k$. At iteration $i+1$, the closest neighbours and consequent rigid transformations $\varvec{R}_{k,i+1}$ are recomputed, and so on, until convergence. *d* is the algorithm’s only parameter, its effect being to control the amount of allowable deformation. Smaller values of *d* permit more deformation and closer alignment of the template to the point cloud, while larger values of *d* favour smooth displacement fields over alignment accuracy. We shall refer to this algorithm using the acronym LAD (Locally Affine Deformation).

In Stradview’s second method, the nonrigid deformation is governed by the SSM, with the template’s vertices constrained according to Eq. (1). The registration again proceeds within an ICP framework. Each of the template’s vertices is paired with the closest point in the cloud. Then, the SSM shape coefficients $S_i$ are found that minimize the sum of the squared distances between the deformed template vertices and their partners in the point cloud. At iteration $i+1$, the closest neighbours and consequent shape coefficients $S_i$ are recomputed, and so on, until convergence. This algorithm is parameter-free, since we use all the available SSM modes in Eq. (1).

### Clinically relevant shape and size measurements

**Figure 4:**
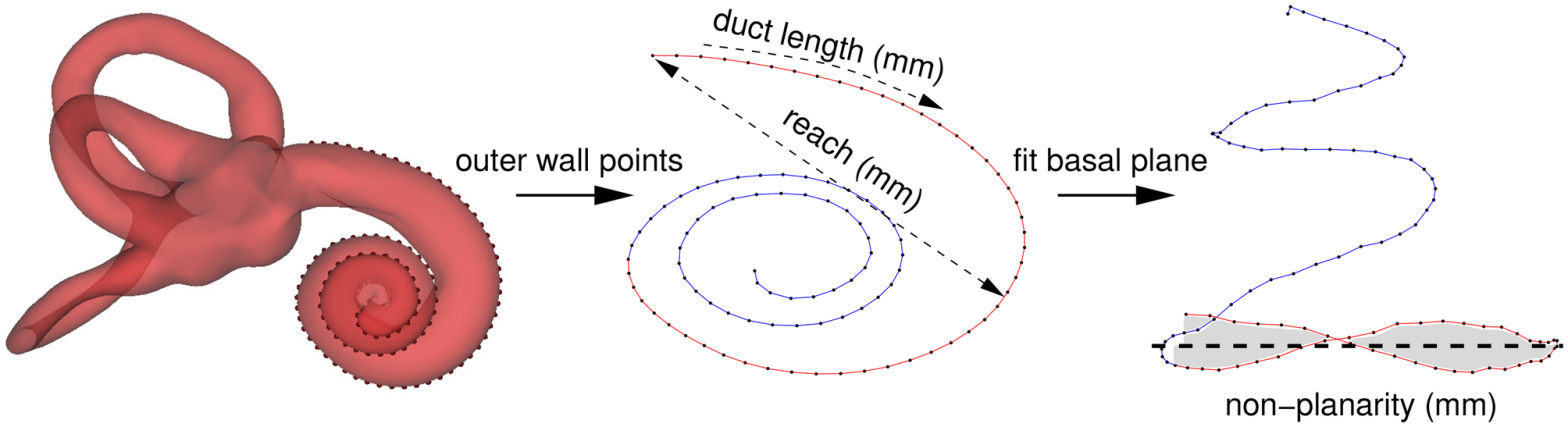
Clinically relevant measurements. In evaluating the success or otherwise of the model fit, we consider the cochlea’s non-planarity, reach and duct length. The non-planarity and reach measurements are made on the first $270^{\circ }$ of the basal turn (red), ignoring the rest of the spiral (blue). Cochlear duct length is measured from the round window to the apex, along the outer (lateral) wall. The basal plane (black dotted line) is defined as the best fit plane to the first $270^{\circ }$ of the outer wall contour. Since we do not detect the modiolus in this study, the $270^{\circ }$ angle is not measured in the usual polar coordinate system defined by the modiolus (origin) and the round window ($0^{\circ }$). Instead, we consider the angle through which the tangent to the outer wall contour has turned with respect to its initial trajectory at the round window. $270^{\circ }$ in this paper’s notation corresponds to somewhat more than $270^{\circ }$ in round window/modiolar polar coordinates.

Figure 4 summarises the three measurements we make on the cochlear surfaces, to compare the similarity of the meshes fitted to pseudo-clinical CT data with their gold-standard counterparts. All measurements are made on the curve that delineates the cochlear outer wall, with particular emphasis on the first $270^{\circ }$ of the basal turn, a range that covers the most common sites of insertion trauma^5–8^. In a one-off process, the outer wall contour was traced on the template mesh by means of a semi-automatic heuristic requiring an expert user to click on points defining the cochlear apex, the centre of the round window and the coiling axis. A contour was then followed automatically from the apex to the round window, passing through those points on the mesh where the surface normal is perpendicular to the coiling axis. The contour was then divided into 100 equal intervals, producing the 101 outer wall points shown in Fig. 4.

**Figure 5:**
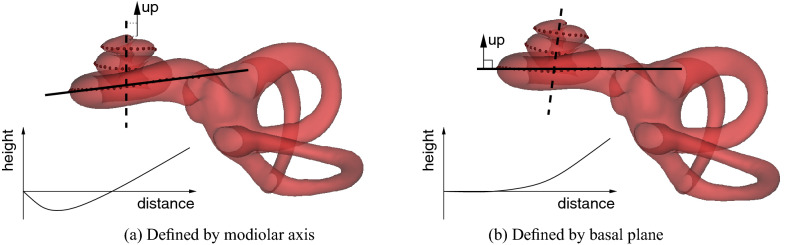
Modiolar and basal coordinates. Previous studies have defined cochlear vertical trajectories with reference to the modiolar axis (dashed line, **a**). However, estimation of this axis is neither straightforward nor (in this context) helpful, since “rollercoaster” height profiles that go down and then up may nevertheless correspond to planar insertion trajectories that present little risk of trauma. An alternative is to replace the modiolar axis with the normal to the best fit plane through the first $270^{\circ }$ of the basal turn (solid line, **b**). In this coordinate system, the “height” axis corresponds to deviation from the best fit plane, and it is clear at which point the insertion trajectory becomes nonplanar and potentially traumatic to the cochlear structures.

The first measurement, which we shall refer to as “reach” (and is comparable with common measures of cochlear size, including the “A-value” of Escudé et al.^13^), is the distance from the round window to the furthest point on the first $270^{\circ }$ of the curve, as defined in Fig. 4. The second is the total cochlear duct length, measured along the outer (lateral) wall, as is common for image-based estimation of this quantity^21^. The third concerns the cochlea’s vertical trajectory, in which a down-then-up “rollercoaster” profile was identified by Avci et al.^15^ as a potential risk factor for insertion trauma. However, Demarcy et al.^35^ observed that vertical trajectories are sensitive to the definition of “vertical”, which is normally taken to be the modiolar axis^15^. We further explore this point in Fig. 5, which shows two vertical trajectories of the *same* cochlea, with the vertical axis defined by the modiolar axis in (a) and the normal to the basal plane in (b). We note not only the sensitivity to the vertical direction, but also that the rollercoaster profile in (a) does not necessarily imply a challenging insertion.

We therefore propose an alternative way to characterize the vertical trajectory. We define the “basal plane” as the best fit plane to the first $270^{\circ }$ of the outer wall contour. Vertical trajectories are measured along the normal to this plane, as in Fig. 5b, thus avoiding any sensitivity to the less germane anatomy of the middle and apical turns. Having established a reliable vertical trajectory, Fig. 4 illustrates how we summarise the “non-planarity” of the basal turn as the mean absolute distance between the first $270^{\circ }$ of the outer wall contour and the basal plane. The hypothesis is that cochleas with lower non-planarity are less susceptible to insertion trauma than those with higher non-planarity.

The consensus approach to cochlear coordinate systems^36^ is somewhat ambiguous, in that the basal plane is assumed to be perpendicular to the modiolus. By anchoring our coordinate system to the basal plane and not the modiolus, we break from the usual interpretation and, unfortunately but inevitably, hinder comparability with previous studies.

## Experiments, results and discussion

The otic capsule model was fitted to each of the 54 pseudo-clinical scans (18 specimens, 3 different resolutions) at grayscale thresholds of 160, 170 and 180. The threshold of 170 was observed to produce visually appropriate segmentations in most cases, with $\pm 10$ re-runs to assess sensitivity. For each data set at each threshold, the model was fitted three times: using the “full” SSM, trained using all 18 micro-CT data sets; using a “leave-one-out” SSM, trained using 17 of the micro-CT data sets, but not the specimen on which it was being evaluated; and using the LAD method, with a fixed parameter $d = 5\,\text {mm}$.

The full SSM provides an upper bound on SSM performance, with an effectively perfect model and fitting compromised only by the image resolution and detector noise. In contrast, the leave-one-out results are indicative of expected performance on unseen specimens with a model trained using only 17 exemplars. Since the LAD method does not require training, the results presented here are expected to generalise to new specimens without gross malformations. The parameter $d = 5\,\text {mm}$ was chosen since it produced visually plausible nonrigid deformations in all cases, without over-fitting to noise. $d = 5\,\text {mm}$ represents a high degree of regularization, as befitting the low-resolution, noisy, pseudo-clinical data: considerably smaller values of *d* would be preferable when fitting to micro-CT data. 50 ICP iterations were used throughout.

**Figure 6:**
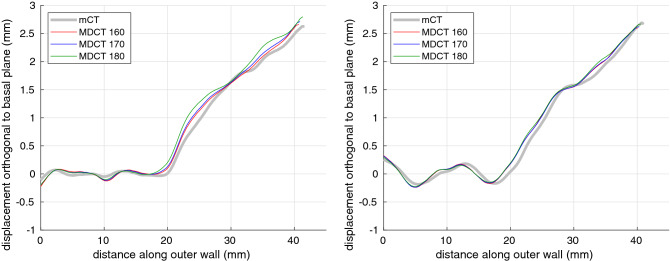
Vertical trajectories of the least (left) and most (right) nonplanar cochleas. The gold-standard micro-CT profiles are displayed in gray. The other profiles are derived from LAD model fits to the standard pseudo-clinical MDCT data, thresholded at 160 (red), 170 (blue) and 180 (green).

**Figure 7:**
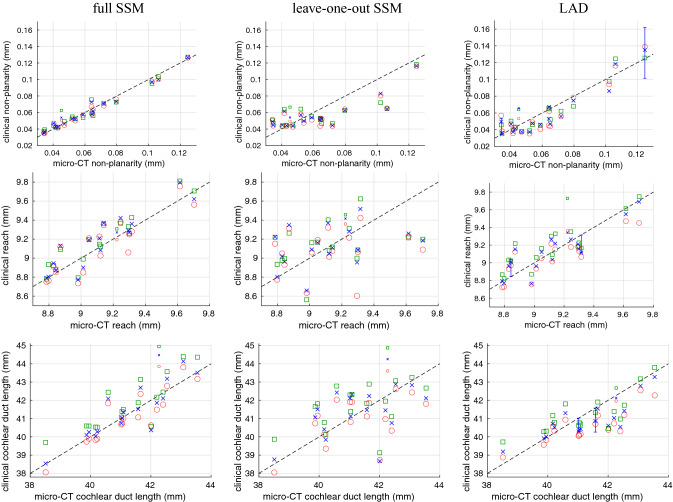
Cochlear non-planarity, reach and duct length estimated from standard pseudo-clinical MDCT. Fully automatic processing with grayscale thresholds of 160 (red circles), 170 (blue crosses) and 180 (green squares). The half-size markers are for the specimen with the implanted electrode. The error bars for the least and most nonplanar cochleas indicate the full range of the results for the LAD sensitivity analyses described in Fig. 8.

Figure 6 illustrates the LAD method’s ability to recover the vertical trajectories of the least and most nonplanar cochleas, when applied to the standard pseudo-clinical MDCT data. Sensitivity to the grayscale segmentation threshold appears to be reasonable. Figure 7 shows the non-planarity, reach and duct length results for standard MDCT, with the gold-standard measurements on the *x*-axis and the measurements derived from the pseudo-clinical images on the *y*-axis. Similar graphs for next-generation CBCT and poor MDCT are omitted here for concision, but may be scrutinized in Gee et al.^37^: they show the expected degradation in performance with lower resolution, more noisy data. Also as expected, the full model performs significantly better than the leave-one-out model. The specimen with the implanted electrode is identified by half-sized markers and is a frequent outlier, since the scans suffered from beam-hardening artefacts that corrupted the thresholded point cloud. Performance might be improved by preprocessing such scans to suppress these artefacts, for instance using the freely available software of Treece^38^, though true clinical scans would be required to test this hypothesis.

The full set of results for all three pseudo-clinical resolutions is summarised in Table 1, omitting the specimen with the implanted electrode. The tabulated numbers are the mean absolute error expressed as a percentage of the gold-standard sample range. Thus, for example, when applied to standard MDCT data, the LAD method produces reach estimates with an expected error of around 12% of the gold-standard range (maximum minus minimum). For comparison, the corresponding errors for the *unfitted* model (i.e. measured directly on the template without fitting to the individual) are 24.0% for non-planarity, 22.9% for reach and 24.1% for duct length.

**Table 1:** Summary results for the 17 specimens without an implanted electrode.

	Threshold	Full model			Leave-one-out model			LAD		
		160	170	180	160	170	180	160	170	180
CBCT	Non-planarity	2.47	2.46	3.35	15.7	16.0	14.8	12.1	10.8	8.63
	Reach	9.36	9.16	9.57	26.7	24.7	23.6	13.0	11.2	10.9
	Duct length	8.35	7.66	13.0	18.5	18.2	20.9	15.0	13.3	12.0
MDCT	Non-planarity	3.04	3.67	4.52	15.1	14.2	15.5	11.7	10.6	8.30
	Reach	13.6	11.2	10.4	26.3	24.1	26.2	13.7	11.5	12.8
	Duct length	10.0	9.30	14.4	19.3	19.1	21.3	16.6	13.2	13.2
Poor MDCT	Non-planarity	4.58	4.61	6.27	16.8	16.0	14.7	17.6	17.4	23.6
	Reach	12.9	9.46	16.9	27.5	28.9	35.4	19.0	17.7	18.1
	Duct length	14.6	12.8	19.2	22.6	21.5	25.7	23.0	20.0	19.1

Average measurement errors are expressed as $100 \; \times$ mean(absolute error)/gold-standard sample range.

Table 2 quantifies the segmentation accuracy of the 17 non-implanted specimens thresholded at a grayscale value of 170. For comparison, the average vertex error for the unfitted model is $0.185\,\text {mm}$. While such results are difficult to interpret from a clinical perspective, they do allow tentative comparison with the work of Noble et al.^22^, who achieved average vertex errors of around $0.2\,\text {mm}$ (fitted) and $0.27\,\text {mm}$ (unfitted). Considerable caution is required though, since Noble et al.^22^ segmented the scala tympani, while Table 2 is for the entire otic capsule. There are also likely differences in the way the gold-standard and clinical coordinate systems were aligned. Kjer et al.^23^ reported mean surface errors of $0.11\,\text {mm}$ for the cochlear scalae.

**Table 2:** Average vertex errors (mm) for the 17 specimens without an implanted electrode, at a grayscale threshold of 170.

	Full model	Leave-one-out model	LAD
CBCT	0.0891	0.144	0.117
MDCT	0.104	0.151	0.123
Poor MDCT	0.120	0.161	0.138

The tabulated numbers are the average distances between vertices on the fitted model and the gold-standard mesh, after optimal rigid body alignment of the two meshes.

On the basis of these results, the LAD approach would appear to offer a practicable way to estimate clinically relevant anatomy of the human cochlea from standard, clinical MDCT. Analysis of one cochlea requires around 1 min of expert interaction followed by several minutes of computation. The expert does need to exercise reasonable care when selecting the segmentation threshold: the one outlying result for the LAD method, for non-planarity at a threshold of 180 with the poor MDCT data, was due to this threshold failing to capture part of the outer wall in many of the scans.

At the central threshold of 170, the LAD approach is able to estimate cochlear reach with a mean absolute error of 11.5% of the gold-standard sample range, or 1.16% ± 0.88% (mean ± one standard deviation) of the gold-standard values. This compares favourably with the method of Iyaniwura et al.^10^, where the absolute error in comparable “A-value” estimates was 2.7% ± 2.1% of the gold-standard values. Kjer et al.^23^ reported signed errors for cochlear size down to $0.02 \pm 0.2 \text {mm}$: our equivalent values for reach are $0.00116 \pm 0.134 \,\text {mm}$. Heutink et al.^26^ reported absolute errors for organ of Corti cochlear duct length of $1.69 \pm 1.13 \text {mm}$: our comparable values at the outer wall are $0.665 \pm 0.495\,\text {mm}$. For the 18 specimens in this study, gold-standard outer wall cochlear duct length range ($38.5{-}43.5\,\text {mm}$) and correlation with reach ($R^2 = 0.39$) are both in good agreement with similar values reported by Erixon and Rask-Andersen^25^.

At the same threshold of 170, cochlear non-planarity was estimated with an average absolute error of 10.6% of the gold-standard sample range. This is a novel metric that we suggest might correlate with the risk of insertion trauma, and may be more reliable than the “rollercoaster” classification of Avci et al.^15^, which is sensitive to estimation of the modiolar axis^35^. While van der Jagt et al.^24^ demonstrated automatic estimation of cochlear vertical trajectories from clinical CT scans, to the best of our knowledge this is the first study to validate such measurements against micro-CT gold standards.

**Figure 8:**
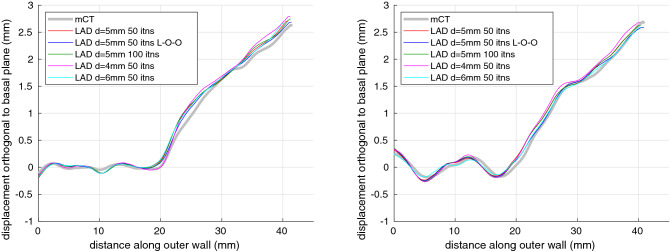
Vertical trajectories of the least (left) and most (right) nonplanar cochleas. The gold-standard micro-CT profiles are displayed in gray. The other profiles are derived from LAD model fits to the standard pseudo-clinical MDCT data thresholded at a grayscale value of 170, with the standard template, an alternative “leave-one-out” (L-O-O) template constructed without the test specimen, different numbers of LAD iterations (itns) and different values of the parameter *d*.

The core LAD experiments in this paper were all conducted with the same template, $d = 5\,\text {mm}$ and 50 ICP iterations, these parameters corresponding to convenient Stradview settings that produced visually plausible deformations and apparently full convergence. Figure 8 and the error bars in Fig. 7 give an indication of parameter sensitivity, using a different template that is independent of each test specimen, double the number of iterations, and both smaller and larger values of *d*. The only material sensitivity appears to be with *d*, where smaller values allow more local deformation to which the non-planarity metric, though not the general form of the vertical trajectory, is sensitive. The metric is a blunt tool for characterizing trajectories, conceivably penalising a series of small, local ripples more than a single, significant hurdle.

A limitation of this study is the focus on just three specific shape and size descriptors. Two other measurements were attempted: lumen area^37^ and the $\phi$ angle between the plumb line of the round window and the tangent to the inner wall of the basal turn^18^, of interest since it constrains the initial insertion and bending angles of the electrode. Neither measurement was successful. For example, at the central threshold of 170, the LAD approach was able to estimate lumen area with a mean absolute error of 18.0% of the gold-standard sample range, which barely improves on the corresponding error of 18.8% for the unfitted model. The prognostic differentiating factor between successful and unsuccessful measurements was involvement of the cochlear inner wall in the latter. Clinical CT contrast at the inner wall is significantly worse than at the outer wall, resulting in poor model fitting around the modiolus. We conclude that anatomical measurements involving the cochlear inner wall are currently infeasible with this methodology.

A further limitation of this study is the use of pseudo-clinical data for the low-resolution model fitting. Real MDCT scans of the temporal bones would arguably have provided a more sound basis for the work, but they were not available. That said, real MDCT data is no panacea: a dissected temporal bone imaged in a clinical MDCT scanner would not appear identical to the same bone scanned intact in a living human being. A reassuring indicator of the validity of the present study is the failure to estimate lumen area or any metric involving identification of the cochlear inner wall.

In comparison with the LAD method, the performance of the SSM approach was disappointing. It was a failure to generalise that limited the SSM’s efficacy in the present study, as evidenced by the relative performance of the full and leave-one-out models. Improved performance may be achievable through more sophisticated grayscale modelling^23^, or by using more training data, or by constraining the shape coefficients to their variation in the training sample, or by limiting the model to the cochlea alone^22,23^. That said, a benefit of including the canals is to leverage the posterior-anterior canal bifurcation as a readily identifiable landmark for initial model positioning.

## Conclusions

We have demonstrated a simple, rapid and freely available technique for estimating cochlear morphology from pseudo-clinical MDCT scans. Average vertex errors are comparable with the state of the art, as are estimates of cochlear size and duct length. A further contribution of this study is an enhanced understanding of the cochlea’s vertical trajectory, leading to a novel metric for characterizing the non-planarity of the basal turn. The non-planarity metric can be estimated from pseudo-clinical scans with an average absolute error of 10.6% of the gold-standard sample range. The hope is that these techniques will perform equally well with true clinical scans, and may one day assist in personalized implant selection and surgical planning, in the same way that similar methods have already been shown to improve implant programming^39^.

## Data Availability

Stradview, wxRegSurf and wxDicom are available for free download: links are provided in the text. The micro-CT scans are not publicly available, since unrestricted publication would breach the terms of the donors’ consent. Reasonable requests for sharing of this data can be made through A.H.G.
